# Neural field models for latent state inference: Application to large-scale neuronal recordings

**DOI:** 10.1371/journal.pcbi.1007442

**Published:** 2019-11-04

**Authors:** Michael E. Rule, David Schnoerr, Matthias H. Hennig, Guido Sanguinetti

**Affiliations:** 1 Department of Engineering, University of Cambridge, Cambridge, United Kingdom; 2 Theoretical Systems Biology, Imperial College London, London, United Kingdom; 3 Department of Informatics, University of Edinburgh, Edinburgh, United Kingdom; Brandeis University, UNITED STATES

## Abstract

Large-scale neural recording methods now allow us to observe large populations of identified single neurons simultaneously, opening a window into neural population dynamics in living organisms. However, distilling such large-scale recordings to build theories of emergent collective dynamics remains a fundamental statistical challenge. The neural field models of Wilson, Cowan, and colleagues remain the mainstay of mathematical population modeling owing to their interpretable, mechanistic parameters and amenability to mathematical analysis. Inspired by recent advances in biochemical modeling, we develop a method based on moment closure to interpret neural field models as latent state-space point-process models, making them amenable to statistical inference. With this approach we can infer the intrinsic states of neurons, such as active and refractory, solely from spiking activity in large populations. After validating this approach with synthetic data, we apply it to high-density recordings of spiking activity in the developing mouse retina. This confirms the essential role of a long lasting refractory state in shaping spatiotemporal properties of neonatal retinal waves. This conceptual and methodological advance opens up new theoretical connections between mathematical theory and point-process state-space models in neural data analysis.

## Author summary

Developing statistical tools to connect single-neuron activity to emergent collective dynamics is vital for building interpretable models of neural activity. Neural field models relate single-neuron activity to emergent collective dynamics in neural populations, but integrating them with data remains challenging. Recently, latent state-space models have emerged as a powerful tool for constructing phenomenological models of neural population activity. The advent of high-density multi-electrode array recordings now enables us to examine large-scale collective neural activity. We show that classical neural field approaches can yield latent state-space equations and demonstrate that this enables inference of the intrinsic states of neurons from recorded spike trains in large populations.

## Introduction

Neurons communicate using electrical impulses, or spikes. Understanding the dynamics and physiology of collective spiking in large networks of neurons is a central challenge in modern neuroscience, with immense translational and clinical potential. Modern technologies such as high-density multi-electrode arrays (HDMEA) enable the simultaneous recording of the electrical activity of thousands of interconnected neurons, promising invaluable insights into neural dynamics at the network level. However, the resulting data is high-dimensional and frequently exhibits complex, non-linear dynamics, presenting formidable statistical challenges.

Due to the complexity of the data, most analyses of neuronal population activity take a descriptive approach, adopting methods from statistical signal processing such as state-space models (SSM; [1–7]) or autoregressive generalized-linear point-process models (PP-GLM; [8–11]). Such methods capture the population statistics of the system, but fail to provide mechanistic explanations of the underlying neural dynamics. While this phenomenological description is valuable and can aid many investigations, the inability to relate microscopic single-neuron properties to emergent collective dynamics limits the scope of these models to extract biological insights from these large population recordings.

Connecting single-neuron dynamics with population behavior has been the central focus of research within the theoretical neuroscience community over the last four decades. Neural field models [12–15] have been crucial in understanding how macroscopic firing dynamics in populations of neurons emerge from the microscopic state of individual neurons. Such models have found diverse applications including working memory (see [16] for a review), epilepsy (e.g. [17–20]), and hallucinations (e.g. [21–23]), and have been successfully related to neuroimaging data such as Electroencepelography (EEG; [24–26]), Magnetoencephelography (MEG; [24]), Electromyography (EMG; [27]), and Functional Magnetic Resonance Imaging (fMRI; [25]), which measure average signals from millions of neurons. Nevertheless, using neural-field models to model HDMEA spiking data directly remains an open statistical problem: HDMEA recordings provide sufficient detail to allow modeling of individual neurons, yet the large number of neurons present prevents the adoption of standard approaches to non-linear data assimilation such as likelihood free inference.

In this paper, we bridge the data-model divide by developing a statistical framework for Bayesian modeling in neural field models. We build on recent advances in stochastic spatiotemporal modeling, in particular a recent result by Schnoerr et al. [28] which showed that a spatiotemporal agent-based model of reaction-diffusion type, similar to the ones underpinning many neural field models, can be approximated as a spatiotemporal point process associated with an intensity (i.e. density) field that evolves in time. Subsequently, Rule and Sanguinetti [29] illustrated a moment-closure approach for mapping stochastic models of neuronal spiking onto latent state-space models, preserving the essential coarse-timescale dynamics. Here, we demonstrate that a similar approach can yield state-space models for neural fields derived directly from a mechanistic microscopic description. This enables us to leverage large-scale spatiotemporal inference techniques [30, 31] to efficiently estimate an approximate likelihood, providing a measure of fit of the model to the data that can be exploited for data assimilation. Our approach is in spirit similar to latent variable models such as the Poisson Linear Dynamical System (PLDS; [5, 32, 33]), with the important difference that the latent variables reflects non-linear neural field dynamics that emerge directly from a stochastic description of single-neuron activity [34–36].

We apply this approach to HDMEA recordings of spontaneous activity from ganglion cells in the developing mouse retina [37], showing that the calibrated model effectively captures the non-linear excitable phenomenon of coordinated, wave-like patterns of spiking [38] that have been considered in both discrete [39] and continuous neural-field models before [40].

## Results

### High level description of the approach

We would like to explain large-scale spatiotemporal spiking activity in terms of the intrinsic states of the participating neurons, which we cannot observe directly. Latent state-space models (SSMs) solve this problem by describing how the unobserved states of neurons relate to spiking observations, and predict how these latent states evolve in time. In this framework, one estimates a distribution over latent states from observations, and uses a forward model to predict how this distribution evolves in time, refining the latent-state estimate with new observations as they become available. This process is often called ‘data assimilation’. However, in order to achieve statistical tractability, SSMs posit simple (typically linear) latent dynamics, which cannot be easily related to underlying neuronal mechanisms. Emergent large-scale spatiotemporal phenomena such as traveling waves typically involve multiple, coupled populations of neurons and nonlinear excitatory dynamics, both of which are difficult to incorporate into conventional state-space models.

Fortunately, mathematical neuroscience has developed methods for describing such dynamics using neural field models. Neural field models map microscopic dynamics to coarse-grained descriptions of how population firing rates evolve. This provides an alternative route to constructing latent state-space models for large-scale spatiotemporal spiking datasets. However, neural field models traditionally do not model statistical uncertainty in the population states they describe, which makes it difficult to deploy them as statistical tools to infer the unobserved, latent states of the neuronal populations. A model of statistical uncertainty is important for describing the uncertainty in the estimated latent states (posterior variance), as well as correlations between states or spatial regions. As we will illustrate, work over the past decades to address noise and correlations in neural field models also provides the tools to employ such models as latent SSMs in data-driven inference.

At a high level then, our approach follows the usual derivation of neural field models, starting with an abstract description of single-neuron dynamics, and considers how population averages evolve in time. Rather than deriving a neural-field equation for the population mean rate, we instead derive two coupled equations for the mean and covariance of population states. We interpret these two moments as a Gaussian-process estimate of the latent spatiotemporal activity, and derive updates for how this distribution evolves in time and how it predicts spiking observations. This provides an interpretation of neural-field dynamics amenable to state-space inference, which allows us to infer neural population states from spiking observations.

### Neural field models for refractoriness-mediated retinal waves

**Fig 1:**
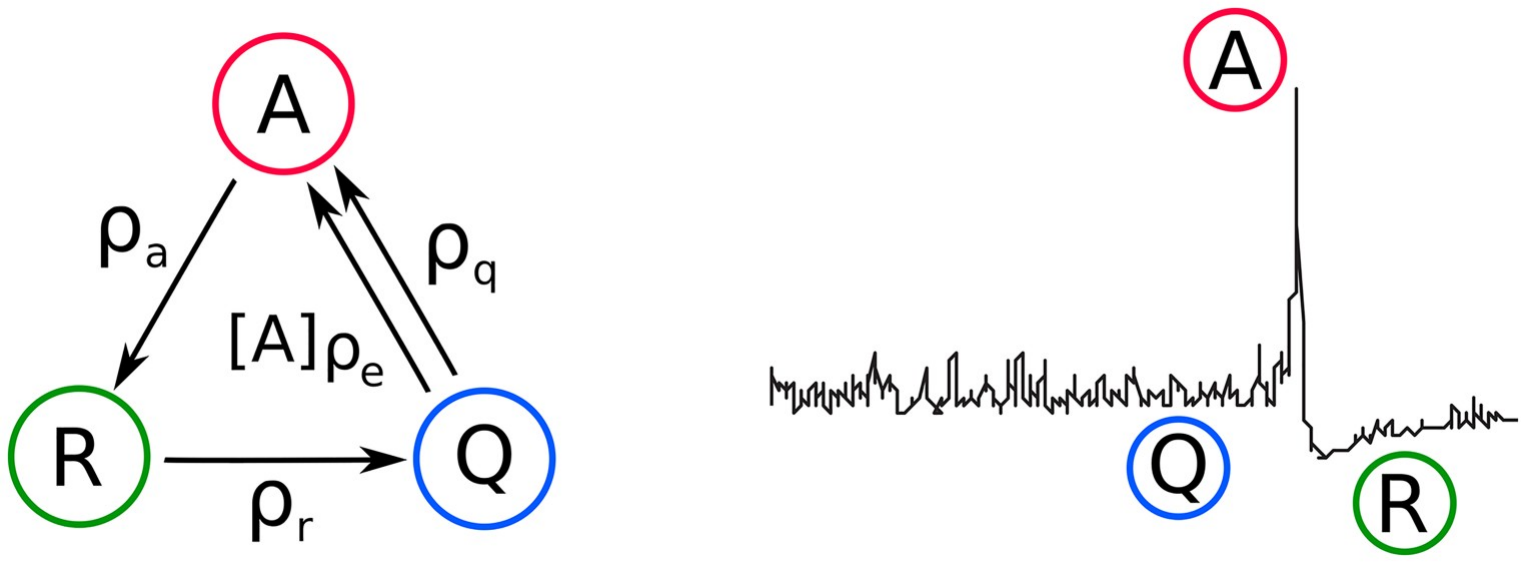
3-state Quiescent-Active-Refractory (QAR) neural-field model. Cells in the developing retina are modeled as having three activity states. Active cells (*A*; red) fire bursts of action potentials, before becoming refractory (*R*; green) for an extended period of time. Quiescent (*Q*; blue) cells may burst spontaneously, or may be recruited into a wave by other active cells. These three states are proposed to underlie critical multi-scale wave dynamics [43].

Although Wilson and Cowan [41, 42] considered refractoriness, most subsequent applications consider only two states: neurons may be either actively spiking (*A* state), or quiescent (*Q* state). In general, voltage and calcium gated conductances typically lead to refractory states, which can be short following individual spikes, or longer after more intensive periods of activity. An excellent example of the importance of a refractory mechanism is found in the developing retina, where a slow afterhyperpolarization (sAHP) current mediates the long-timescale refractory effects that strongly shapes the spatiotemporal dynamics of spontaneous retinal waves [43]. To address this, we explicitly incorporate additional refractory (*R*) states into our neural field model (e.g. [44, 45]; Fig 1). In the following, we first outline a non-spatial model for such system, before extending it to a spatial setting with spatial couplings. Finally, we develop a Bayesian inference scheme for inferring latent states from observational data.

### A stochastic three-state neural mass model

We now consider the neural field model with three states as a generic model of a spiking neuron (Fig 1), where a neuron can be in either an actively spiking (A), refractory (R), or quiescent (Q) state. We assume that the neurons can undergo the following four transitions:

$$\begin{array}{c} \begin{array}{cccccc} Q & \overset{\rho_{q}}{\to }A & & & Q+A & \overset{\rho_{e}}{\to }A+A\\ A & \overset{\rho_{a}}{\to }R & & & R & \overset{\rho_{r}}{\to }Q, \end{array} \end{array}$$ (1)

i.e. quiescent neurons transition spontaneously to the active state; active neurons excite quiescent neurons; active neurons become refractory, and refractory neurons become quiescent. The *ρ*_(⋅)_ denote corresponding rate constants.

**Fig 2:**
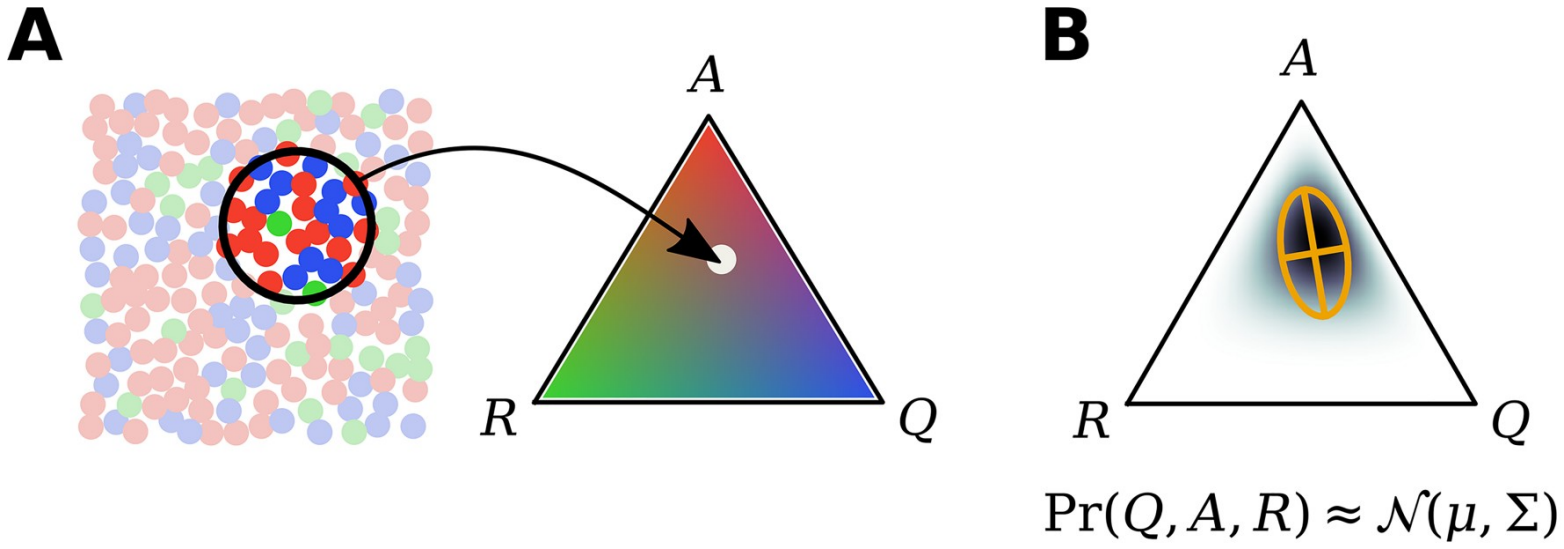
Summarizing estimated neural state as population moments. **(A)** The activity within a local spatial region (encircled, left) can be summarized by the fraction of cells (represented by colored dots) in the quiescent (blue), active (red), and refractory (green) states (*Q*, *A*, *R*; right). **(B)** An estimate of the population state can be summarized as a probability distribution Pr(*Q*, *A*, *R*) over the possible proportions of neurons in each state. A Gaussian moment-closure approximates this distribution as Gaussian, with given mean and covariance (orange crosshairs).

For illustration, we first consider the dynamics of a local (as opposed to spatially-extended) population of neurons. In this case the state of the system is given by the non-negative number counts *Q*, *A* and *R* of the respective neuron types (we slightly abuse notation here and use *Q*, *A*, and *R* both as symbols for the neuron states and as variables counting the neurons in the corresponding states; see Fig 2 for an illustration). The time evolution of the corresponding probability distribution to be in a state (*Q*, *A*, *R*) at a certain time point is then given by a master equation ([34, 44, 46]; Methods: *Moment-closure for a single population*). Due to the nonlinear excitatory interaction *Q* + *A* → *A* + *A* in Eq (1), no analytic solutions to the master equation are known. To get an approximate description of the dynamics, we employ the Gaussian moment closure method which approximates the discrete neural counts (*Q*, *A*, *R*) by continuous variables, and assumes a multivariate normal distribution (Fig 2B; [29, 34, 35, 47–50]). This allows one to derive a closed set of ordinary differential equations for the mean and covariance of the approximate process which can be solved efficiently numerically (Methods: *Moment-closure for a single population*; Fig 2).

Applying this procedure to our system leads to the following evolution equations of the first moments (mean concentrations):

$$\begin{array}{cc} \partial_{t}\langle Q\rangle =r_{rq}-r_{qa} & r_{qa}=\rho_{q}\langle Q\rangle +\rho_{e}\langle AQ\rangle \\ \partial_{t}\langle A\rangle =r_{qa}-r_{ar} & r_{ar}=\rho_{a}\langle A\rangle \\ \partial_{t}\langle R\rangle =r_{ar}-r_{rq} & r_{rq}=\rho_{r}\langle R\rangle , \end{array}$$ (2)

where the rate variables *r*_(⋅)(⋅)_ describe the rates of the different transitions in Eq (1), and 〈⋅〉 denotes expected-value with respect to the distribution over population states. Intuitively, Eq (2) says that the mean number of neurons in each state evolves according to the difference between the rate that neurons enter, and the rate that neurons leave, said state. For spontaneous (Poisson) state transitions, these rates are linear and depend only on the average number of neurons in the starting state. The transition from *Q* to *A*, however, has both a spontaneous and excito-excitatory component. The latter depends on the expected product of active and quiescent cells 〈*AQ*〉, which is a second moment and can be expressed in terms of the covariance: 〈*AQ*〉 = 〈*A*〉〈*Q*〉 + Σ_*AQ*_. We obtain similar equations for the covariance of the system (Eq 6; Methods: *Moment-closure for a single population*). These can be solved jointly with Eq (2) forward in time to give an approximation of the system’s dynamics.

### Generalization to spatial (neural field) system

So far we have considered a single local population. We next extend our model to a two-dimensional spatial system. In this case the mean concentrations become density or mean fields (‘neural fields’) that depend on spatial coordinates **x** = (*x*_1_, *x*_2_), e.g. 〈*Q*〉 becomes 〈**Q**(**x**)〉. Similarly, the covariances become two-point correlation functions. For example, Σ_*QA*_(**x**, **x**′) denotes the covariance between the number of neurons in the quiescent state at location **x** and the number of neurons in the active state at location **x**′ (see Methods: *Extension to spatial system* for details).

By replacing the mean concentrations and covariances accordingly in Eqs (2) and (6), we obtain spatial evolution equations for these space-dependent quantities. The terms arising from the linear transitions in Eq (1) (i.e. *r*_*rq*_, *r*_*aq*_ and the first term in *r*_*qa*_ in Eq 2) do not introduce any spatial coupling and hence do not need to be modified (note also that neurons do not diffuse or move otherwise, which is why we do not obtain a dynamic term in the resulting equations). The nonlinear excitatory interaction *Q* + *A* → *A* + *A* in Eq (1), however, introduces a coupling which we need to specify further in a spatial setting. We assume that each quiescent neuron experiences an excitatory drive from nearby active neurons, and that the interaction strength can be described as a function of distance ||Δ**x**|| by a Gaussian interaction kernel:

$$\begin{array}{c} \begin{array}{c} k\left(\Delta x\right)\propto \exp\left(-{\left||\Delta x|\right|}^{2}/2\sigma_{e}^{2}\right), \end{array} \end{array}$$ (3)

where *σ*_*e*_ the standard deviation determining the length scale of the interaction, which decays exponentially as a function of distance squared. This kernel introduces a spatial coupling between the neurons, which could be mediated by synaptic interactions, diffusing neurotransmitters, gap junction coupling, or combinations thereof. With this coupling, the transition rate (compare to Eq (2)) from the quiescent to active state at position **x** becomes the following integral:

$$\begin{array}{c} \begin{array}{cc} r_{qa}\left(x\right) & =\rho_{q}\left\langle Q\left(x\right)\right\rangle +\rho_{e}\int k\left(x-x^{\prime }\right)\left\langle Q\left(x\right)A\left(x^{\prime }\right)\right\rangle dx^{\prime }, \end{array} \end{array}$$ (4)

where the integral runs over the whole volume of the system (Methods: *Extension to spatial system*).

**Fig 3:**
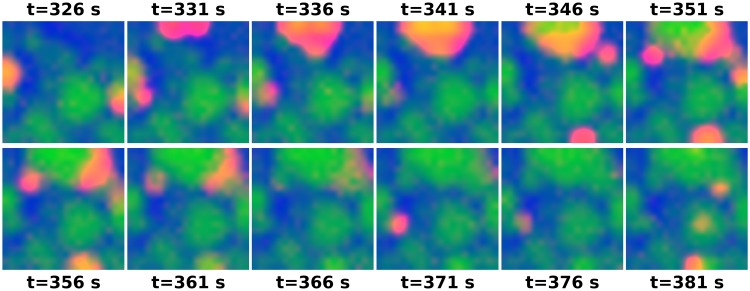
Spatial 3-state neural-field model exhibits self-organized multi-scale wave phenomena. Simulated example states at selected time-points on a [0, 1]^2^ unit interval using a 20 × 20 grid with effective population density of *ρ* = 50 cells per unit area, and rate parameters *σ* = 0.075, *ρ*_*a*_ = 0.4, *ρ*_*r*_ = 3.2 × 10^−3^, *ρ*_*e*_ = 0.028, and *ρ*_*q*_ = 0.25 (Methods: *Sampling from the model*). As, for instance, in neonatal retinal waves, spontaneous excitation of quiescent cells (blue) lead to propagating waves of activity (red), which establish localized patches in which cells are refractory (green) to subsequent wave propagation. Over time, this leads to diverse patterns of waves at a range of spatial scales.

We thus obtain a ‘second-order’ neural field in terms of the mean fields and two-point correlation functions. We simulated the spatially-extended system by sampling. Fig 3 shows that it is indeed capable of producing multi-scale wave-like phenomena similar to the waves observed in the retina (Methods: *Sampling from the model*).

### Neural field models as latent-variable state-space models

**Fig 4:**
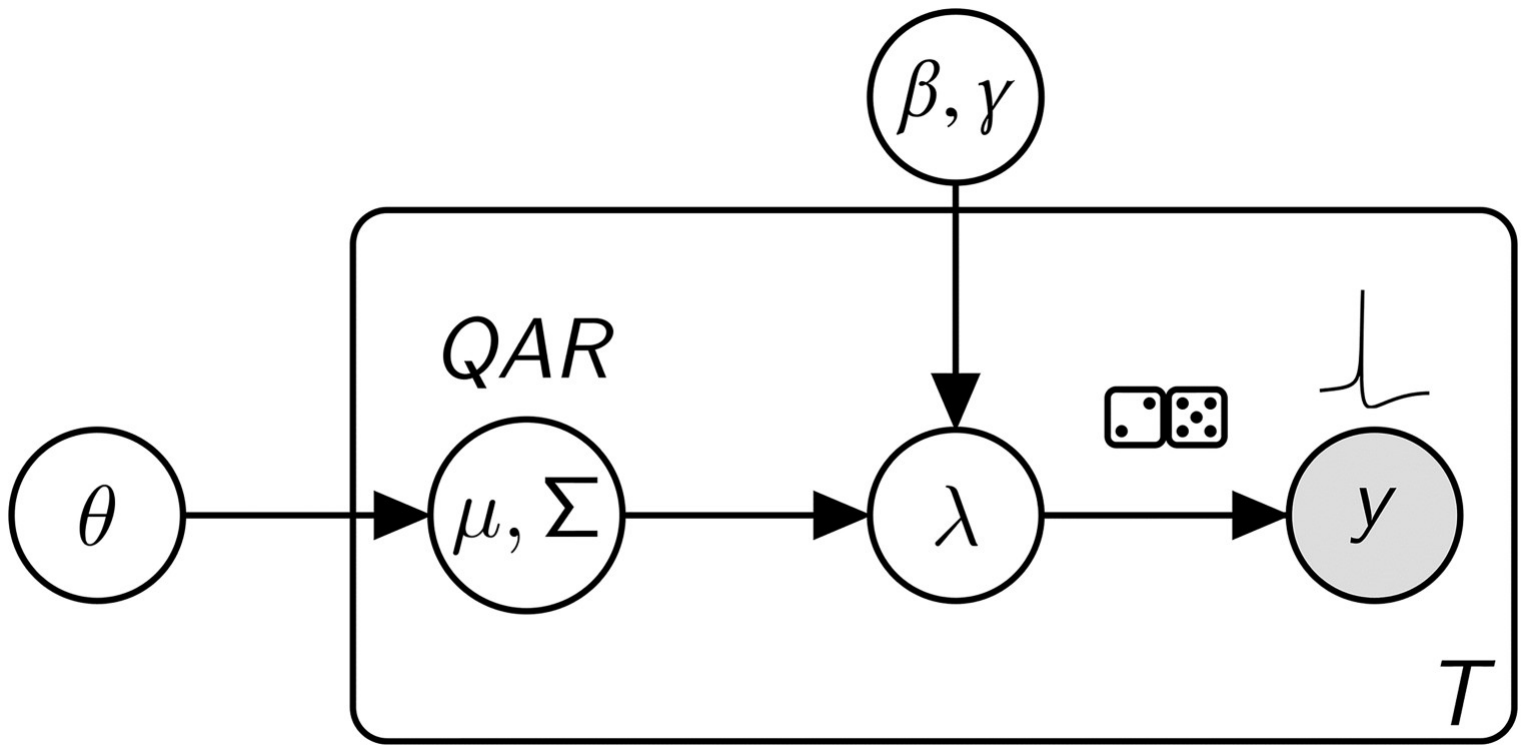
Hidden Markov model for latent neural fields. For all time-points *T*, state transition parameters *θ* = (*ρ*_*q*_, *ρ*_*a*_, *ρ*_*r*_, *ρ*_*e*_, *σ*) dictate the evolution of a multivariate Gaussian model *μ*, Σ of latent fields *Q*, *A*, *R*. The observation model (*β*, *γ*) is a linear map with adjustable gain and threshold, and reflects how field *A* couples to firing intensity λ. Point-process observations (spikes) *y* are Poisson with intensity λ.

The equations for the mean fields and correlations can be integrated forward in time and used as a state-space model to explain population spiking activity (Fig 4; Methods: *Bayesian filtering*). In extracellular recordings, we do not directly observe the intensity functions 〈**Q**(**x**)〉, 〈**A**(**x**)〉, and 〈**R**(**x**)〉. Instead, we observe the spikes that active neurons emit, or in the case of developmental retinal waves recorded via a HDMEA setup, we observe the spikes of retinal ganglion cells which are driven by latent wave activity. The spiking intensity should hence depend on the density **A**(**x**) of active neurons. Here, we assume that neural firing is a Poisson process conditioned on the number of active neurons, which allows us to write the likelihood of point (i.e. spike) observations in terms of **A**(**x**) ([10, 11, 51]; Methods: *Point-process measurement likelihood*).

The combination of this Poisson-process observation model with the state-space model derived in previous sections describes how hidden neural field states evolve in time and how these states drive neuronal spiking. Given spatiotemporal spiking data, the latent neural field states and correlations can then be inferred using a sequential Bayesian filtering algorithm. The latter uses the neural field model to predict how latent states evolve, and updates this estimate at each time point based on the observed neuronal spiking (Methods: *Bayesian filtering*). This provides estimates of the unobserved physiological states of the neurons.

**Fig 5:**
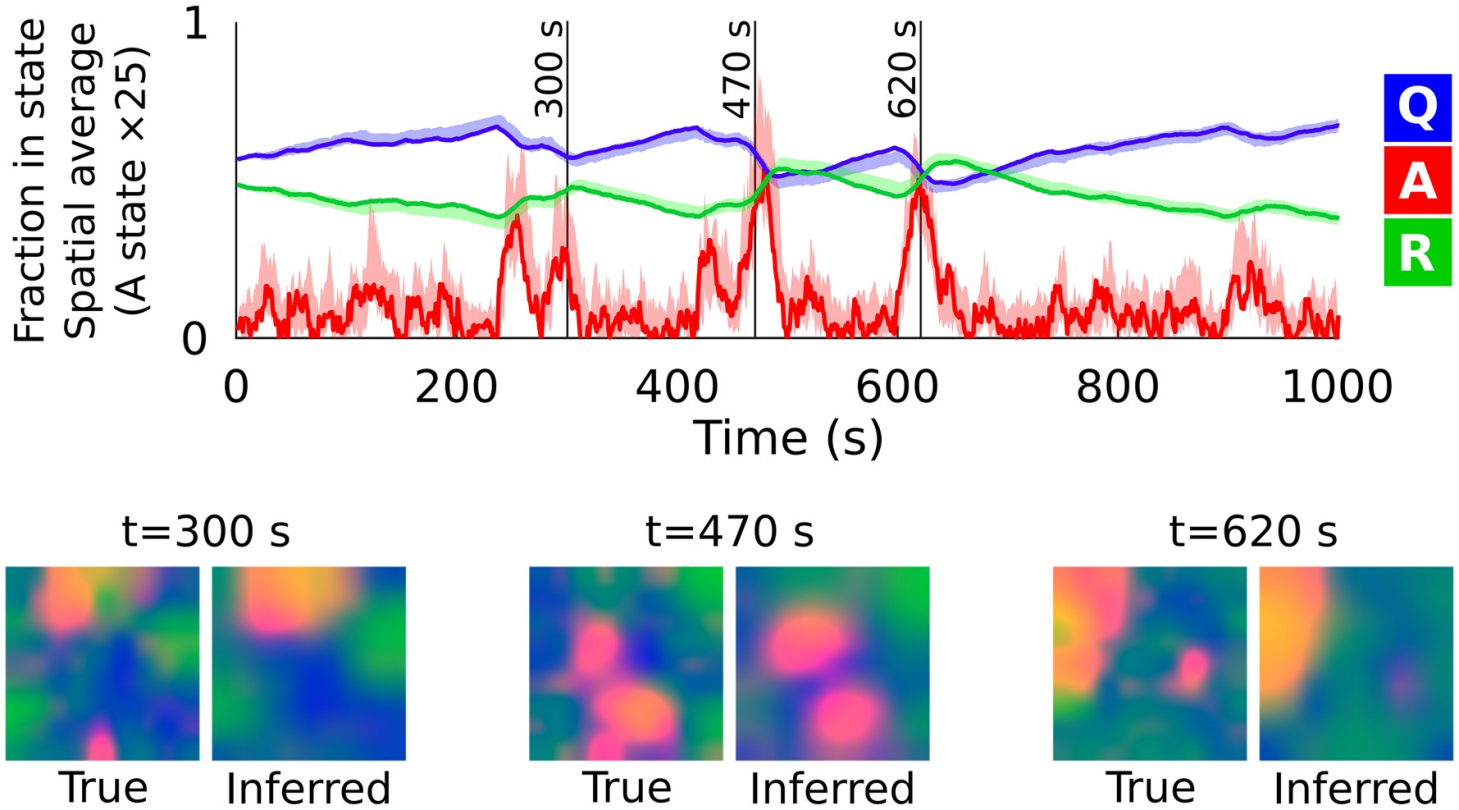
Filtering recovers latent states in ground-truth simulated data. Spatially averaged state occupancy (blue, red, and green: **Q**, **A**, and **R**) (vertical axis) is plotted over time (horizontal axis). Solid lines represent true values sampled from the model, and shaded regions represent the 95% confidence interval estimated by filtering. The active (**A**) state density has been scaled by ×25 for visualization. Colored plots (below) show the qualitative spatial organization of quiescent (blue), active (red), and refractory (green) neurons. Model parameters are the same as Fig 3, with the exception of the spatial resolution, which has been reduced to 9 × 9.

We verified that this approach works using simulated data. We first simulated observations from the neural field equations (Fig 3; Methods: *Sampling from the model*), which generated waves qualitatively similar to those seen in the developing retina. We then sampled spiking as a conditionally-Poisson process driven by the number of active neurons in each location, with a baseline rate of *β* = 0 and gain of *γ* = 15 spikes/second per simulation area. We then applied Bayesian filtering to these spiking samples in order to recover a Gaussian estimate of the latent neural field states (Methods: *Bayesian filtering*). Fig 5 illustrates the latent states recovered via filtering using the known ground-truth model parameters, and shows that filtering can recover latent neural field states from the spiking observations. Overall, this indicates that moment-closure of stochastic neural field equations can yield state-space models suitable for state inference from spiking data. In the next section, we illustrate this approach applied to waves recorded from the developing retina.

### State inference in developmental retinal waves

**Fig 6:**
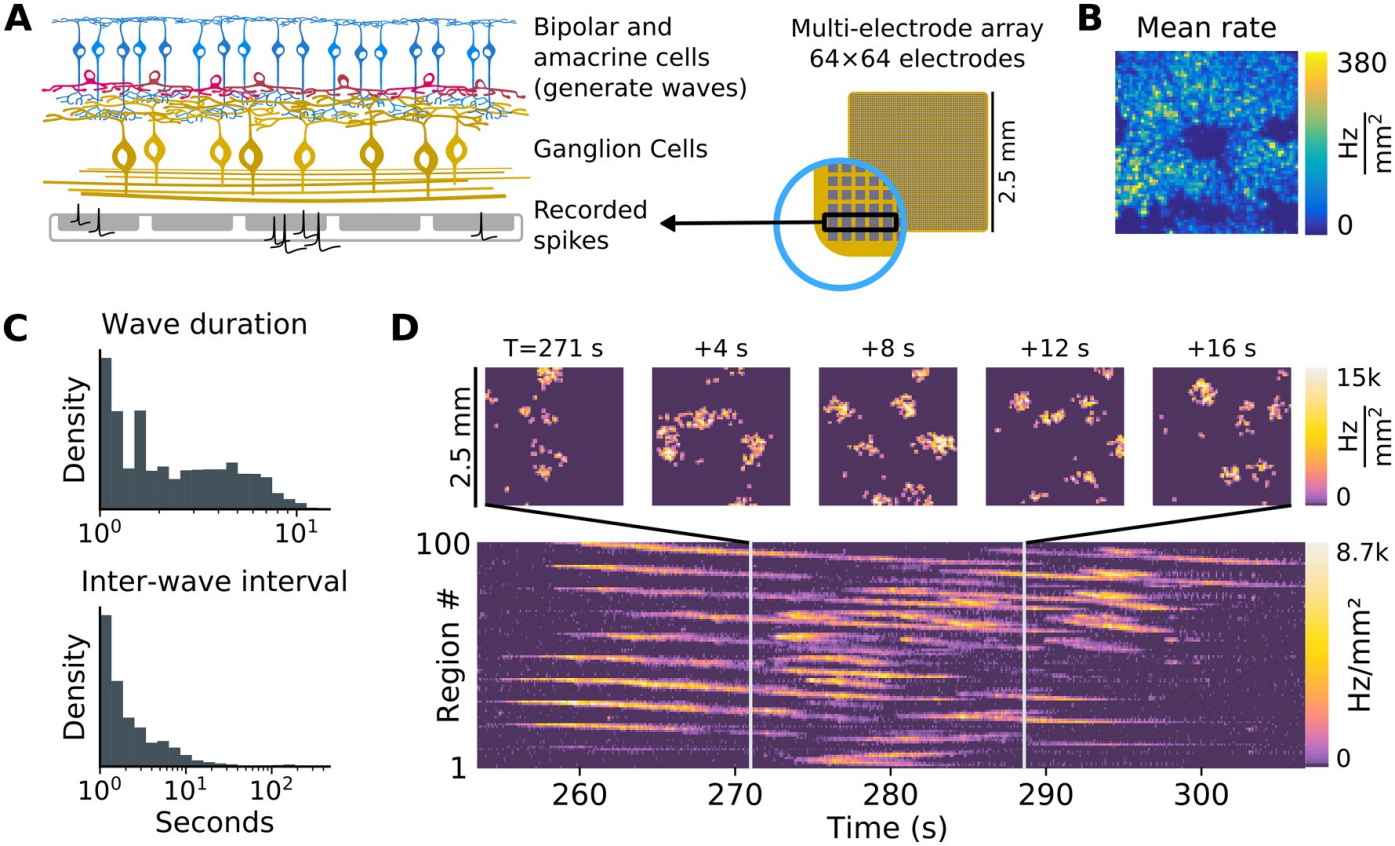
Retinal waves recorded via high-density multi-electrode arrays. **(A)** Spontaneous retinal waves are generated in the inner retina via laterally interacting bipolar (blue) and amacrine (red) cells, depending on the developmental age. These waves activate retinal ganglion cells (yellow), the output cells of the retina. Electrical activity is recorded from the neonatal mouse retina via a 64 × 64-electrode array with 42 μm spacing. **(B)** Average spiking rate recorded across the retina (the central region devoid of recorded spikes is the optic disc). This example was recorded on postnatal day 6. **(C)** Spikes were binned at 100 ms resolution, and assigned to 10 × 10 spatial regions for analysis. Spiking activity on each electrode was segmented into “up” states (during wave activity) and “down” states (quiescent) using a two-state hidden Markov model with Poisson observations. In this example, most waves and inter-wave intervals lasted between one and ten seconds. **(D)** Example wave event, traveling across multiple spatial regions and lasting for a duration of 16-20 seconds.

Having developed an interpretation of neural field equations as a latent-variable state-space model, we next applied this model to the analysis of spatiotemporal spiking data from spontaneous traveling wave activity occurring in the neonatal vertebrate retina (e.g. Fig 6; [37–39, 52–55]).

During retinal development, the cell types that participate in wave generation change [37, 52, 54], but the three-state model globally describes dynamics in the inner retina at all developmental stages (Fig 6). The Active (*A*) state describes a sustained bursting state, such as the depolarization characteristic of starburst amacrine cells (Fig 6a) during acetylcholine-mediated early-stage (Stage 2) waves between P0 and P9 [54, 55], and late-stage (Stage 3) glutamate-dependent waves [54, 56]. For example, Fig 6c and 6d illustrates spontaneous retinal wave activity recorded from a postnatal day 6 mouse pup (Stage 2). In addition, at least for cholinergic waves, the slow refractory state *R* is essential for restricting wave propagation into previously active areas [57]. We note that the multi-scale wave activity exhibited in the three-state neural field model (e.g. Fig 3) recapitulates the phenomenology of retinal wave activity explored in the discrete three-state model of Hennig et al. [43].

**Fig 7:**
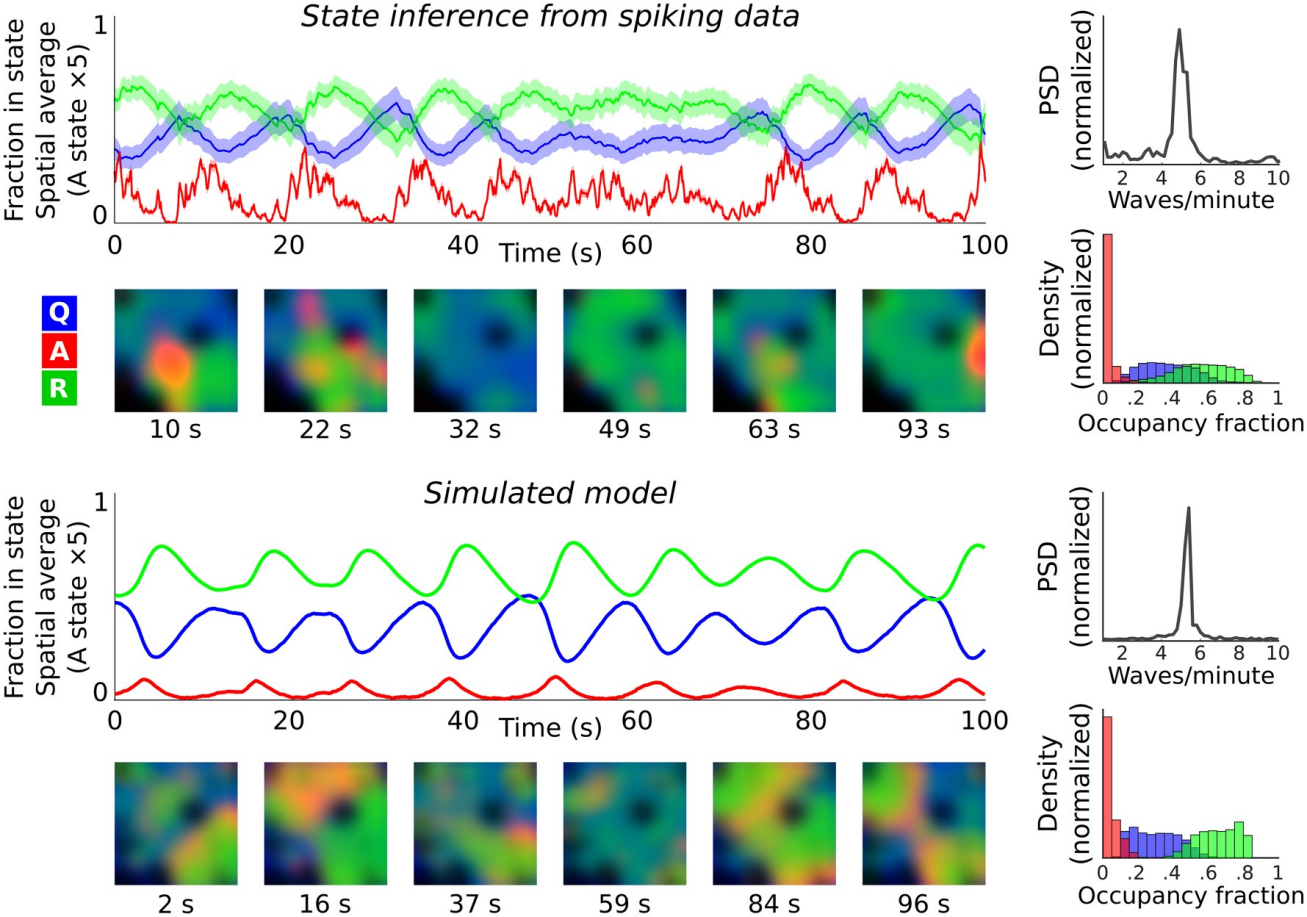
State inference via filtering: Retinal datasets. We apply a calibrated model to spiking observations from retinal waves (postnatal day 11) to infer latent neural-field states. In all plots, red, green, and blue indicate (normalized) densities of active, refractory, and quiescent cells. **(top)** Solid lines indicate inferred spatial means, and shaded regions the 95% confidence bound. The the *A* state has been scaled-up by ×5. Example time slices are shown in the colored plots below. Dark regions indicate areas absent from the recording. Summary statistics are shown on the right, with power spectra (averaged over all included regions and states) indicating periodic ∼5 waves/min, and the typical fraction of Q/A/R states, pooled over all times and regions, summarized in histograms below. **(bottom)** Forward simulation of the calibrated model without data recapitulates the retinal wave activity. Solid lines indicate sampled spatial means. Colored plots show example time slices. Wave frequency is comparable to the data (∼5 waves/min), and occupancy statistics are similar. The model was initialized with 70% of cells quiescent and 30% refractory, with a 25 s burn-in to remove initial transients.

Using RGC spikes recorded with a 4,096-electrode HDMEA (Fig 6), we demonstrate the practicality of latent-state inference using heuristic rate parameters and illustrate an example of inference for a retinal wave dataset from postnatal day 11 (Stage 3; Fig 7). For retinal wave inference, we normalize the model by population-size (Methods: *System-size scaling*) so that the gain and bias do not depend on the local neuronal population size.

The state inference (‘data assimilation’) procedure uses new observations to correct for prediction errors. Because of this, many different model parameters may give similar state estimates. Nevertheless, it is important that the rate parameters approximately match the data. The rate of excitation (*ρ*_*e*_) should be fast, and the rate at which active cells become refractory (*ρ*_*a*_) should match the typical wave duration. Likewise, it is important that the recovery rate *ρ*_*r*_ matches the inter-wave interval timescale. In Fig 7, model parameters were set based on observed timescales, and then adjusted such that the simulated model dynamics match those recovered during state inference (*ρ*_*e*_ = 10, *ρ*_*a*_ = 1.8 *ρ*_*r*_ = 0.1, and *σ* = 0.1). These parameters were held fixed during subsequent state inference. The interaction radius *σ* = 0.15 and excitation strength *ρ*_*e*_ interact to determine how excitable the system is and how quickly waves propagate. The overall excitability should be small enough so that the system is stable, and does not predict wave events in the absence of spiking observations. As in Lansdell et al. [40], lateral interactions in our model reflect an effective coupling that combines both excitatory synaptic interactions and the putative effect of diffusing excitatory neurotransmitters, which has been shown to promote late-stage glutamatergic wave propagation [53].

The moment-closure system does not accurately approximate the rare and abrupt nature of wave initiation. We therefore model spontaneous wave-initiation events as an extrinsic noise source, and set the spontaneous excitation rate *ρ*_*q*_ to zero in the neural field model that defines our latent state-space. The Poisson noise was re-scaled to reflect an effective population size of 16 neurons/mm², significantly smaller than the true population density [58]. However, due to the recurrent architecture and correlated neuronal firing, the effective population size is expected to be smaller than the true population size. Equivalently, this amounts to assuming supra-Poisson scaling of fluctuations for the neural population responsible for retinal waves.

Bayesian filtering recovers the expected features of the retinal waves (Fig 7): the excito-excitatory transition *Q* + *A* → *A* + *A* and the onset of refractoriness *A* → *R* are rapid compared to the slow refractory dynamics, and therefore the *A* state is briefly occupied and mediates an effective *Q* → *R* transition during wave events. The second-order structure provided by the covariance is essential, as it allows us to model posterior variance (shaded regions in Fig 7), while also capturing strong anti-correlations due to the conservation of reacting agents, and the effect of correlated fluctuations on the evolution of the means. Furthermore, spatial correlations allow localized RGC spiking events to be interpreted as evidence of regional (spatially-extended) latent neuronal activity.

### Open challenges in model identification

So far, we have demonstrated good recovery of states when the true rate parameters are known (Fig 5), and shown that plausible latent-states can be inferred from neural point-process datasets using *a priori* initialized parameters (Fig 7). A natural question then is whether one can use the Bayesian state-space framework to estimate a posterior likelihood on the rate parameter values, and infer model parameters directly from data. Presently, model inference remains challenging for four reasons: under-constrained parameters, computational complexity, numerical stability, and non-convexity in the joint posterior. It is worth reviewing these challenges as they relate to important open problems in machine learning and data assimilation.

*First*, the effective population size, the typical fraction of units in quiescent vs. refractory states, and the gain parameter mapping latent activations to spiking, are all important to setting appropriate rates, and are not accessible from observation of RGC spiking alone. Recovering a physiologically realistic model would require direct measurement or appropriate physiological priors on these parameters. In effect, many equivalent systems can explain the observed RGC spiking activity, a phenomenon that has been termed “sloppiness” in biological systems [59, 60]. Indeed, Hennig et al. [61] show that developmental waves are robust to pharmacological perturbations, suggesting that the retina itself can use different configurations to achieve similar wave patterns. *Second*, although state inference is computationally feasible, parameter inference requires many thousands of state-inference evaluations. A Matlab implementation of state-inference running on a 2.9 GHz 8-core Xeon CPU can process ∼85 samples/s for a 3-state system on a 10 × 10 spatial basis. For a thirty-minute recording of retinal wave activity, state inference is feasible, but repeated state inference for parameter inference is impractical. *Third*, model likelihood must be computed recursively, and is subject to loss of numerical accuracy due to back-propagation through time [62–64]. In other words, small errors in the past can have large effects in the future owing to the nonlinear and excitable nature of the system. *Fourth* and finally, the overall likelihood surface need not be convex, and may contain multiple local optima. Additionally, regions of parameters space can exhibit vanishing gradient for one or model parameters. This can occur when the value of one parameter makes others irrelevant. For example, if the excito-excitatory interaction *ρ*_*e*_ is set to a low value, the interaction radius *σ*_*e*_ for excitation becomes irrelevant since the overall excitation is negligible.

Overall, parameter inference via Bayesian filtering presents a formidable technical challenge. Presently, it seems that traditional methods, based on mathematical expertise and matching observable physical quantities (e.g. wavefront speed, c.f. [40]), remain the best-available approach to model estimation. Despite these challenges, the innovation presented here, of applying moment-closure methods for data assimilation, is important *per se*, because it provides a snapshot of the activity of unobserved states which can greatly aid scientific investigation. The state-space formulation of neural field models enables Bayesian state inference from candidate neural field models, and opens the possibility of likelihood-based parameter inference in the future.

## Discussion

In this work, we showed that classical neural-field models, which capture the activity of large, interacting neural populations, can be interpreted as state-space models, where we can explicitly model microscopic, intrinsic dynamics of the neurons. This is achieved by interpreting a second-order neural field model as defining equations on the first two moments of a latent-variable process, which is coupled to spiking observations. In the state-space model interpretation, latent neural field states can be recovered from Bayesian filtering. This allows inferring the internal states of neuronal populations in large networks based solely on recorded spiking activity, information that can experimentally only be obtained with whole cell recordings.

We demonstrated successful state inference for simulated data, where the correct model and parameters were known. Next, we applied the model to large-scale recordings of developmental retinal waves. Here the correct latent-state model is unknown, but a relatively simple three-state model with slow refractoriness is well-motivated by experimental observations [57]. Previous works [39, 57, 65, 66] predict that activity-dependent refractoriness is important for restricting the spatial spreading of waves. Intuitively, one should expect the refractory time constant to be a highly sensitive parameter: very long refractory constants will impede the formation of waves, while short constants might lead to interference phenomena. These intuitions were borne out empirically by our simulation studies; additionally, we observed that long refractory constants led to ineffective data assimilation, as the model prior is too dissimilar from the data it is trained upon. In contrast to phenomenological latent state-space models, the latent states here are motivated by an (albeit simplified) description of single-neuron dynamics, and the state-space equations arise directly from considering the evolution of collective activity as a stochastic process.

In the example explored here, we use Gaussian moment-closure to arrive at a second-order approximation of the distribution of latent states and their evolution. In principle, other distributional assumptions may also be used to close the moment expansion. Other mathematical approaches that yield second-order models could also be employed, for example the linear noise approximation [67], or defining a second cumulant in terms of the departure of the model from Poisson statistics [35]. The approach applied here to a three-state system can generally be applied to systems composed of linear and quadratic state transitions. Importantly, systems with only linear and pairwise (quadratic) interactions can be viewed as a locally-quadratic approximation of a more general smooth nonlinear system [68], and Gaussian moment closure therefore provides a general approach to deriving approximate state-space models in neural population dynamics.

The state-space interpretation of neural field models opens up future work to leverage the algorithmic tools of SSM estimation for data assimilation with spiking point-process datasets. However, challenges remain regarding the retinal waves explored here, and future work is needed to address these challenges. Model likelihood estimation is especially challenging. Despite this, the connection between neural-field models and state-space models derived here will allow neural field modeling to incorporate future advances in estimating recursive, nonlinear, spatiotemporal models. We also emphasize that some of the numerical challenges inherent to high-dimensional spatially extended neural field models do not apply to simpler, low-dimensional neural mass models, and the moment-closure framework may therefore provide a practical avenue to parameter inference in such models.

In summary, this report connects neural field models, which are grounded in models of stochastic population dynamics, to latent state-space models for population spiking activity. This connection opens up new approaches to fitting neural field models to spiking data. We expect that this interpretation is a step toward the design of coarse-grained models of neural activity that have physically interpretable parameters, have physically measurable states, and retain an explicit connection between microscopic activity and emergent collective dynamics. Such models will be essential for building models of collective dynamics that can predict the effects of manipulations on single-cells on emergent population activity.

## Materials and methods

### Data acquisition and preparation

Example retinal wave datasets are taken from Maccione et al. [37]. Spikes were binned at 100 ms resolution for analysis. Spikes were further binned into regions on a 20 × 20 spatial grid. For the three-state model, this resulted in a 1200-dimensional spatiotemporal system, which provided an acceptable trade-off between spatial resolution and numerical tractability.

Spiking activity in each region was segmented into wave-like and quiescent states using a two-state hidden Markov model with a Poisson observations. To address heterogeneity in the Retinal Ganglion Cell (RGC) outputs, the observation model was adapted to each spatial region based on firing rates. Background activity was used to establish per-region biases, defined as the mean activity in a region during quiescent periods. The scaling between latent states and firing rate (gain) was adjusted locally based on the mean firing rate during wave events. The overall (global) gain for the observation model was then adjusted so that no wave events exhibited a fraction of cells in the active (*A*) state greater than one.

### Moment-closure for a single population

To develop a state-space formalism for inference and data assimilation in neural field models, we begin with a master equation approach. This approach has been used before to analyze various stochastic neural population models, often as a starting point to derive ordinary differential equations for the moments of the distribution of population states, as we do here [34–36, 44, 46, 69]. In our case, we examine a three-state system of the kind proposed in Buice and Cowan [44, 45], and use a Gaussian moment-closure approach similar to Bressloff [34].

The master equation describes how the joint probability distribution of neural population states (in our example the active, quiescent and refractory states) evolves in time. However, modelling this full distribution is computationally prohibitive for a spatially-extended system, since the number of possible states scales exponentially with the number of neural populations. Instead, we approximate the time evolution of the moments of this distribution.

In principle, an infinite number of moments are needed to describe the full population activity. To limit this complexity, we consider only the first two moments (mean and covariance), and use a moment-closure approach to close the series expansion of network interactions in terms of higher moments ([47–50]; for applications to neuroscience see [29, 34–36, 69, 70]). Using this strategy, we obtain a second-order neural field model that describes how the mean and covariance of population spiking evolve in time, and recapitulates spatiotemporal phenomena when sampled.

We may describe the number of neurons in each state in terms of a probability distribution Pr(*Q*, *A*, *R*) (Fig 2A), where we slightly abuse notation and use *Q*, *A*, and *R* both as symbols for the neuron states and as variables counting the neurons in the corresponding states, i.e. non-negative integers. The time evolution of this probability distribution captures stochastic population dynamics, and is represented by a master equation that describes the change in density for a given state {*Q*, *A*, *R*} when neurons change states. Accordingly, the master equation describes the change in probability of a given state {*Q*, *A*, *R*} in terms of the probability of entering, minus the probability of leaving the state:

$$\begin{array}{cccc} \partial_{t}\mathrm{Pr}\left(Q,A,R\right) & =\mathrm{Pr}\left(Q,A+1,R-1\right)\rho_{a}\left(A+1\right) & & \text{(transition}\;A\to R\text{)}\\ & +\mathrm{Pr}\left(Q-1,A,R+1\right)\rho_{r}\left(R+1\right) & & \text{(transition}\;R\to Q\text{)}\\ & +\mathrm{Pr}\left(Q+1,A-1,R\right)[\rho_{q}+\rho_{e}\left(A-1\right)]\left(Q+1\right) & & \text{(}Q\to A\;\text{and}\;A+Q\to A+A\text{)}\\ & -\mathrm{Pr}\left(Q,A,R\right)[\left(\rho_{\text{e}}A+\rho_{\text{q}}\right)Q+\rho_{\text{a}}A+\rho_{\text{r}}R] & & \text{(outgoing}\;\text{transitions)} \end{array}$$ (5)

Even in this simplified non-spatial scenario, no analytic solutions are known for the master equation. However, from Eq (5) one can derive equations for the mean and covariance of the process.

The approach, generally, is to consider expectations of individual states, e.g. 〈*Q*〉 (first moments, i.e. means), or 〈*QA*〉 (second moments), taken with respect to the probability distribution Pr(*Q*, *A*, *R*) described by the master Eq (5). Differentiating these moments in time, and substituting in the time-evolution of the probability density as given by the master equation, yields expressions for the time-evolution of the moments. However, in general these expressions will depend on higher moments and are therefore not closed.

For our system, the nonlinear excitatory interaction *Q* + *A* → *A* + *A* couples the evolution of the means to the covariance Σ_*AQ*_, and the evolution of the covariance is coupled to the third moment, and so on. The moment equations are therefore not closed, and require an infinite number of moments to describe the evolution of the mean and covariance. To address this complexity, we approximate Pr(*Q*, *A*, *R*) with a multivariate normal distribution at each time-point (Fig 2B), thereby replacing counts of neurons with continuous variables. This Gaussian moment-closure approximation sets all cumulants beyond the variance to zero, yielding an expression for the third moment in terms of the mean and covariance, leading to closed ordinary differential equations for the means and covariances [47–50].

For our model with transitions given in Eq (1) this leads to the system of ODEs for the mean values given in Eq (2) in the main text. For the evolution of the covariance we obtain

∂tΣ=JΣ+ΣJT+Σnoise,Σnoise=[rqa+rrq-rqa-rrq-rqarqa+rar-rar-rrq-rarrar+rqa]J=[-ρq-ρe⟨A⟩-ρe⟨Q⟩ρr-ρq+ρe⟨A⟩ρe⟨Q⟩-ρa00ρa-ρr] (6)

where **J** is the Jacobian of the equations for the deterministic means in Eq (2), and the Σ_noise_ fluctuations are Poisson and therefore proportional to the mean reaction rates (Eq 2). Intuitively, the Jacobian terms **J** describe how the covariance of the state distribution ‘stretches’ or ‘shrinks’ along with the deterministic evolution of the means, and the additional Σ_noise_ reflects added uncertainty due to the fact that state transitions are stochastic. Each state experiences Poisson fluctuations with variance equal to the mean transition rates, due to the sum of transitions into and away from the state. Because the number of neurons is conserved, a positive fluctuation into one state implies a negative fluctuation away from another, yielding off-diagonal anticorrelations in the noise.

Together, Eqs (2) and (6) provide approximate equations for the evolution of the first two moments of the master equation (Eq 5), expressed in terms of ordinary differential equations governing the mean and covariance of a multivariate Gaussian distribution. Here, we have illustrated equations for a 3-state system, but the approach is general and can be applied to any system with spontaneous and pairwise state transitions.

### Extension to spatial system

To extend the moment Eqs (2) and (6) to a neural field system, we consider a population of neurons at each spatial location. In this spatially-extended case, we denote the intensity fields as **Q**, **A**, and **R**, which are now vectors with spatial indices (or, in the spatially-continuous case: scalar functions of coordinates **x**). In the spatially-extended system, active (**A**) neurons can excite nearby quiescent (**Q**) neurons. We model the excitatory influence of active cells as a weighted sum over active neurons in a local neighborhood, defined by a coupling kernel *K*(Δ**x**) that depends on distance (Eq 4). To simplify the derivations that follow, denote the convolution integral in Eq (4) as a linear operator **K** such that

$$\begin{array}{c} KA=K(\Delta x)*A(x). \end{array}$$ (7)

In this notation, one can think of **K** as a matrix that defines excitatory coupling between nearby spatial regions. Using the notation of Eq (7), the rate that active cells excite quiescent ones is given by the product

$$\begin{array}{c} \rho_{e}\left(KA\right)○Q=\rho_{e}\mathrm{Diag}(KAQ^{⊤}), \end{array}$$ (8)

where ○ denotes element-wise (in the spatially-continuous case: function) multiplication. For the time evolution of the first moment (mean intensity) of **Q** in the spatial system, one therefore considers the expectation 〈**KAQ**^⊤^〉, as opposed to 〈*AQ*〉 in the non-spatial system. Since **K** is a linear operator, and the extension of the Gaussian state-space model over the spatial domain **x** is a Gaussian process, the second moment of the nonlocal interactions **KA** with **Q** can be obtained in the same way as one obtains the correlation for a linear transformation of a multivariate Gaussian variable:

$$\begin{array}{c} \begin{array}{cc} \left\langle KAQ^{⊤}\right\rangle & =K\langle AQ^{⊤}\rangle \\ & =K(\Sigma_{A,Q}+\left\langle A\right\rangle {\left\langle Q\right\rangle }^{⊤}). \end{array} \end{array}$$ (9)

The resulting equations for the spatial means are similar to the nonspatial system (Eq 2), with the exception that we now include spatial coupling in the rate at which quiescent cells enter the active state:

$$\begin{array}{c} \begin{array}{cc} r_{qa} & =\rho_{q}\left\langle Q\right\rangle +\rho_{e}\mathrm{Diag}[\langle KAQ^{⊤}\rangle ]\\ & =\rho_{q}\left\langle Q\right\rangle +\rho_{e}\mathrm{Diag}[K(\Sigma_{A,Q}+K\left\langle A\right\rangle {\left\langle Q\right\rangle }^{⊤})]\\ & =\rho_{q}\left\langle Q\right\rangle +\rho_{e}[\mathrm{Diag}(K\Sigma_{A,Q})+K\left\langle A\right\rangle ○\left\langle Q\right\rangle ]. \end{array} \end{array}$$ (10)

The numbers of neurons in the quiescent verses active states are typically anti-correlated, because a neuron entering the active state implies that one has left the quiescent state. Therefore, the expected number of interactions between quiescent and active neurons is typically smaller than what one might expect from the deterministic mean field alone. The influence of correlations Diag(**K**Σ_**A**,**Q**_) on the excitation is therefore important for stabilizing the excitatory dynamics.

To extend the equations for the second moment to the neural field case, we consider the effect of spatial couplings on the Jacobian (Eq 6). The spontaneous first-order reactions remain local, and so the linear contributions are similar to the non-spatial case. However, nonlocal interaction terms emerge in the nonlinear contribution to the Jacobian:

Jnonlinear=ρe︷GradientinQ︷GradientinA0[-Diag(K⟨A⟩)-Diag(⟨Q⟩K)0-Diag(K⟨A⟩)-Diag(⟨Q⟩K)0000], (11)

where here the “Diag” operation refers to constructing a diagonal matrix from a vector. Intuitively, the first column of Eq (11) reflects the fact that the availability of quiescent cells modulates the excitatory effect of active cells, and the second column reflects the fact that the density active of neurons in nearby spatial volumes contribute to the rate at which quiescent cells become active.

### Basis projection

The continuous neural field equations are simulated by projection onto a finite spatial basis *B*. Each basis element is an integral over a spatial volume. Means for each basis element are defined as an integral over this volume, and correlations are defined as a double integral. For example, consider the number of quiescent neurons associated with the *i*^*th*^ basis function *B*_*i*_, which we will denote as *Q*_*i*_. The mean 〈*Q*_*i*_〉 and covariance $\Sigma_{QA}^{ij}$ between the quiescent and active states are given by the projections:

$$\begin{array}{c} \begin{array}{cc} \left\langle Q_{i}\right\rangle & =\int B_{i}\left(x\right)Q\left(x\right)\;dx\\ \Sigma_{QA}^{ij} & =\;\int \int \;B_{i}\left(x\right)B_{j}\left(x^{\prime }\right)\Sigma_{QA}\left(x,x^{\prime }\right)\;dx\;dx^{\prime }, \end{array} \end{array}$$ (12)

where **x** and **x**′ range over spatial coordinates as in Eqs (3) and (4). When selecting a basis *B*, assumptions must be made about the minimum spatial scale to model. A natural choice is the radius of lateral (i.e. spatially nonlocal) interactions in the model *σ*_*e*_ (Eq 3), since structure below this scale is attenuated by the averaging over many nearby neurons in the dendritic inputs.

### Sampling from the model

For ground-truth simulations, we sample from a hybrid stochastic model derived from a Langevin approximation to the three-state neural field equation. In this approximation, the deterministic evolution of the state is given by the mean-field equations (Eq (2) for a local system, Eq (10) for the neural field system), and the stochastic noise arising from Poisson state transitions is approximated as Gaussian as given by second-order terms (i.e. Σ_noise_ in Eq (6); see also [50, 71]). Spontaneous wave initiation events are too rare to approximate as Gaussian, and instead are sampled as Poisson (shot) noise, giving us a hybrid stochastic model:

$$\begin{array}{c} \begin{array}{cc} r_{q}\left(t\right) & ∼\mathrm{Poisson}\left(\rho_{q}\cdot dt\right)\cdot \delta \left(t\right), \end{array} \end{array}$$ (13)

where *δ*(*t*) is a Dirac delta (impulse). To avoid uniform spontaneous excitation, the excito-excitatory reaction rate is adjusted by a small finite threshold *ϑ*, i.e. *r*_*qa*_ ← max(0, *r*_*qa*_ − *ϑ*) in Eq (10). For our simulations (e.g. Fig 3), we let *ϑ* = 8 × 10^−3^. For the non-spatial system, the hybrid stochastic differential equation is:

[dQdAdR]=([-rq(t)0-ρr-rq(t)-ρa00-ρa-ρr][QAR]+ρe[-QA-QA0])dt+Σnoise1/2dW, (14)

where Σ_noise_ is the fluctuation noise covariance as in Eq (6) (with *ρ*_*q*_ excluded, as it is addressed by the shot noise, Eq 13), and *dW* is the derivative of a multidimensional standard Wiener process, i.e. a spherical (white) Gaussian noise source. The deterministic component of (14) equation can be compared to Eq (2) for the means of the non-spatial system in the moment-closure system (without the covariance terms).

The stochastic differential equation for the spatial system is similar, consisting to a collection of local populations coupled through the spatial interaction kernel (Eqs 3 and 4), and follows the same derivation used when extending the moment-closure to the spatial case (Methods: *Extension to spatial system*, Eqs 7–10). When applying the Euler-Maruyama method method to the spatiotemporal implementation, fluctuations were scaled by $\sqrt{\Delta t\;\Delta x}$, where Δ**x** is the volume of the spatial basis functions used to approximate the spatial system (see Methods: *System-size scaling* for further detail). The Euler-Maruyama algorithm samples noise from a Gaussian distribution, and can therefore create negative intensities due to discretization error. We addressed this issue by using the complex Langevin equation [72], which accommodates transient negative states.

### Point-process measurement likelihood

Similarly to generalized linear point-process models for neural spiking [10, 11, 51], we model spikes as a Poisson process conditioned on a latent intensity function *λ*(**x**, *t*), which characterises the probability of finding a certain number of spikes *k* in a small spatiotemporal interval Δ*x* × Δ*t* as:

$$\begin{array}{c} \mathrm{Pr}(\int_{t_{0}}^{t_{0}+\Delta t}\int_{x_{0}}^{x_{0}+\Delta x}y\left(x,t\right)\;dx\;dt=k)∼\mathrm{Poisson}(k;\int_{t_{0}}^{t_{0}+\Delta t}\int_{x_{0}}^{x_{0}+\Delta x}\lambda \left(x,t\right)\;dx\;dt). \end{array}$$ (15)

In (15), *y*(**x**, *t*) denotes the experimentally-observed spiking output, and is a sum over Dirac delta distributions corresponding to each spike with an associated time *t*_*i*_ and spatial location **x**_*i*_, i.e. *y*(**x**, *t*) = ∑_*i*∈1..*N*_ *δ*(**x**_*i*_)*δ*(*t*_*i*_). We use a linear Poisson likelihood for which the point-process intensity function

$$\begin{array}{c} \lambda (x,t)=\gamma (x)A(x,t)+\beta (x) \end{array}$$ (16)

depends linearly on the number of active neurons **A**(**x**, *t*) with spatially-varying gain *γ*(**x**) and bias *β*(**x**). In other words, the observed firing intensity in a given spatiotemporal volume should be proportional to the number of active neurons, with some additional offset or bias *β* to capture background spiking unrelated to the neural-field dynamics.

### Bayesian filtering

Having established an approach to approximate the time-evolution of the moments of a neural field system, we now discuss how Bayesian filtering allows us to incorporate observations in the estimation of the latent states. Suppose we have measurements *y*_0_, …, *y*_*N*_ of the latent state *x* at time *t*_0_, …, *t*_*N*_, given by a measurement process $\mathrm{Pr}(y_{i}|x_{t_{i}})$, which in our case is given by the point-process likelihood (Eq 16). Bayesian filtering allows us to recursively estimate the *filtering distribution* $\mathrm{Pr}(x_{t_{i}}|y_{i},\ldots ,y_{0})$ at time *t*_*i*_, i.e. the posterior state probability at time *t*_*i*_ given the current and all previous observations. The procedure works by the following iterative scheme: i) suppose we know the filtering distribution $\mathrm{Pr}(x_{t_{i}}|y_{i},\ldots ,y_{0})$ at time *t*_*i*_. Solving the model dynamics forward in time up to *t*_*i*+1_ gives the predictive distribution Pr(*x*_*t*_|*y*_*i*_, …, *y*_0_) for all times *t*_*i*_ < *t* ≤ *t*_*t*+1_. ii) at the time *t*_*i*+1_ the measurement *y*_*i*+1_ needs to be taken into account which can be done by means of the Bayesian update:

$$\begin{array}{c} \mathrm{Pr}\left(x_{i+1}|y_{i+1},\ldots ,y_{0}\right)=\frac{\mathrm{Pr}\left(y_{i+1}|x_{i+1}\right)\mathrm{Pr}\left(x_{i+1}|y_{i},\ldots ,y_{0}\right)}{\mathrm{Pr}(y_{i+1}|y_{i},\ldots ,y_{0})}, \end{array}$$ (17)

where we have used the Markov property and Pr(*y*_*i*+1_|*x*_*i*+1_, *y*_*i*_, …, *y*_0_) = Pr(*y*_*i*+1_|*x*_*i*+1_) to obtain the right hand side. Eq (17) gives the filtering $\mathrm{Pr}(x_{t_{i+1}}|y_{i+1},\ldots ,y_{0})$ at time *t*_*i*+1_ which serves as the input of the next *i* step. Performing steps i) and ii) iteratively hence provides the filtering distribution for all times *t*_0_ ≤ *t* ≤ *t*_*n*_.

For our neural field model we must compute both steps approximately: to obtain the predictive distribution in step i) we integrate forward the differential equations for mean and covariance derived from moment-closure (Eqs 2–6 and Methods: *Extension to spatial system*). In practice, we convert the continuous-time model to discrete time. If *F*_∂*t*_ denotes the local linearization of the mean dynamics in continuous time such that ∂_*t*_ *μ*(*t*) = *F*_∂*t*_ *μ*(*t*), then the approximated discrete-time forward operator is

$$\begin{array}{c} F_{\Delta t}=\exp\left(F_{\partial t}\Delta t\right)\approx I+F_{\partial t}\Delta t. \end{array}$$ (18)

We update the covariance using this discrete-time forward operator, combined with an Euler integration step for the Poisson fluctuations. A small constant diagonal regularization term Σ_reg_ can be added, if needed, to improve stability. The resulting equations read:

$$\begin{array}{c} \begin{array}{cc} \mu_{t+\Delta |t} & =F_{\Delta t}\mu_{t}\\ \Sigma_{t+\Delta |t} & =F_{\Delta t}\Sigma_{t}F_{\Delta t}^{T}+\Sigma_{t}^{\text{noise}}\cdot \Delta t+\Sigma_{\text{reg}}. \end{array} \end{array}$$ (19)

This form is similar to the update for a discrete-time Kalman filter [73, 74], the main difference being that the dynamics between observation times are taken from the nonlinear moment equations.

Consider next the measurement update of step ii) in Eq (17). Since the Gaussian model for the latent states *x* is not conjugate with the Poisson distribution for observations *y*, we approximate the posterior Pr(*x*_*i*+1_|*y*_*i*+1_, …, *y*_0_) using the Laplace approximation (c.f. [1, 32]). The Laplace-approximated measurement update is computed using a Newton-Raphson algorithm. The measurement update is constrained to avoid negative values in the latent fields by adding a *ε*/*x* potential (compare to the log-barrier approach; [27]), which ensures that the objective function gradient points away from this constraint boundary, where *x* is the intensity of any of the three fields. The gradients and Hessian for the posterior measurement log-likelihood lnL are

$$\begin{array}{c} \begin{array}{cc} -\ln L & =\frac{1}{2}{\left(x-\mu \right)}^{T}\Sigma^{-1}\left(x-\mu \right)+v\left(\gamma x+\beta \right)-y\ln\left(\gamma x+\beta \right)\\ -\frac{\partial \ln L}{\partial x} & =\Sigma^{-1}\left(x-\mu \right)+v\gamma -y(\frac{\gamma }{\gamma x+\beta })\\ -\frac{\partial^{2}\ln L}{\partial x^{2}} & =\Sigma^{-1}+y{\left(\frac{\gamma }{\gamma x+\beta }\right)}^{2}, \end{array} \end{array}$$ (20)

where *x* is the latent state with prior mean *μ* and covariance Σ, and couples to point-process observations *y* linearly with gain *γ* and bias *β* as in Eq (16). The parameter *v* = Δ*x*^2^ ⋅ Δ*t* is the spatiotemporal volume of the basis function or spatial region over which the counts are observed.

### System-size scaling

For clarity, the derivations in this paper are presented for a population of neurons with a known size, such that the fields **Q**(**x**), **A**(**x**), and **R**(**x**) have units of *neurons*. In practice, the population size Ω of neurons is unknown, and it becomes expedient to work in normalized intensities, where **Q**(**x**), **A**(**x**), and **R**(**x**) represent the *fraction* of neurons in a given state between 0 and 1, and are constrained such that **Q**(**x**) + **A**(**x**) + **R**(**x**) = 1. In this normalized model for population size Ω, quadratic interaction parameters (like *ρ*_*e*_) as well as the gain are multiplied by Ω, to reflect the re-scaled population. In contrast, noise variance should be *divided* by Ω to account for the fact that the coefficient of variation decreases as population size increases. Although rescaling by Ω is well-defined for finite-sized populations, the infinitesimal neural-field limit for the second-order model is not. This is because, while the mean-field equations scale with the population size O(Ω), the standard deviation of Poisson fluctuations scales with the square root of the population size $O(\sqrt{\Omega })$. The ratio of fluctuations to the mean (coefficient of variation) therefore scales as $O(1/\sqrt{\Omega })$, which diverges as Ω → 0.

This divergence is not an issue in practice as all numerical simulations are implemented on a set of basis functions with finite nonzero volumes, and each spatial region is therefore associated with finite nonzero population size. Even in the limit where fluctuations would begin to diverge, one can treat the neural field equations as if defined over a continuous set of overlapping basis functions with nonzero volume. Conceptually, this can be viewed as setting a minimum spatial scale for the neural field equations, which is defined by spatial extent of each local population. If the model is defined over a set of overlapping spatial regions, then these populations experience correlated fluctuations. Consider Poisson fluctuations as entering with some rate-density *σ*^2^(**x**) per unit area. The observed noise variances and covariances, projected onto basis functions *B*_*i*_(**x**) and *B*_*j*_(**x**), are:

$$\begin{array}{c} \begin{array}{cc} \Sigma_{i,j}^{\text{noise}} & =\int B_{i}\left(x\right)B_{j}\left(x\right)\sigma^{2}\left(x\right)dx \end{array} \end{array}$$ (21)

If the neuronal population density is given as *ρ*(**x**) per unit area, then the effective population size for a given basis function is:

$$\begin{array}{c} \begin{array}{cc} \Omega_{i} & =\int B_{i}\left(x\right)\rho \left(x\right)dx \end{array} \end{array}$$ (22)

If the population density is uniform, and if basis functions have a constant volume *v*, we can write this more simply as Ω = *vρ*. In the system-size normalized model, the contributions of basis function volume cancel and the noise variance should be scaled simply as 1/*ρ*.
